# On the Availibility of Certain Antiseptics in the Prophylactic Treatment of the Oral Cavity

**Published:** 1885-07

**Authors:** W. B. Miller

**Affiliations:** Berlin.


ARTICLE II.

### ON THE AVAILIBILITY OF CERTAIN ANTISEPTICS IN THE PROPHYLACTIC TREATMENT OF THE ORAL CAVITY.

BY PROF. W. B. MILLER, A. B., D. D. S. BERLIN.

In the *Independent Practitioner* (page 283—1884), and *Dental Record* (page 341—1884), I published a table giving the results of a series of experiments on the antiseptic action of a considerable number of medicaments used in the treatment of the oral cavity. A few new materials have since been added, and I here produce the complete table.

| Antiseptic | Concentration necessary to prevent development of micro-organisms. |
|---|---|
| Bichloride of Mercury . . | 1 — 100,000 |
| Nitrate of Silver . . . . . | 1 — 50,000 |
| Peroxide of Hydrogen* . . | 1 — 8,000 |
| Iodine . . . . . . . | 1 — 6,000 |
| Iodoform . . . . . . | 1 — 5,000 |
| Naphthaline . . . . . | 1 — 4,000 |
| Salicylic Acid (Crystals)* . . | 1 — 2,000 |
| Benzoic Acid* . . . . . | 1 — 1,500 |
| Permanganate of Potash. . . | 1 — 1,000 |
| Eucalyptus Oil . . . . | 1 — 600 |
| Carbolic Acid . . . . . | 1 — 500 |
| Hydrochloric Acid . . . . | 1 — 500 |
| Biborate of Soda* . . . . . | 1 — 350 |
| Arsenious Acid* . . . . | 1 — 250 |
| Chloride of Zinc . . . . . | 1 — 250 |
| Lactic Acid . . . . . | 1 — 125 |
| Carbonate of Sodium . . . . | 1 — 100 |
| Listerine* . . . . . . | 1 — 20 |
| Alcohol . . . . . . . | 1 — 10 |
| Chlorate of Potash* . . . . | 1 — 8 |

Those substances designated by a * were tested upon a pure culture of one of the most common ferment organisms of the mouth, the others were tested upon mixtures. In the majority of cases a stronger solution will be necessary to sterilise (or prevent development in) a mixture than a pure culture, and where solid substances are present, as in the saliva, (particles of food, &c.), the fungi resist still longer the action of the antiseptic.

It must furthermore be readily apparent to every one that the real value of these materials must not be set down as proportional to the numbers in the table above. To say that the bichloride of mercury is, for dental uses, 200 times as valuable as carbolic acid would be as little admissible as to say that dynamite is, in proportion to its greater power, more valuable for industrial purposes than horses.

The question of the availability of different forms of energy, which has long been one of greatest interest to physicists, merits also in the study of antiseptics a thorough consideration. A partial solution of this question I have endeavored to present in the following notes. I have employed in each case the highest concentration that may be readily used in the human mouth as a wash or on the brush.

The substances tested will be found in the following table:

The 1 per cent. solutions of benzoic and salicylic acids (in 20 per cent. alcohol) are rather too sharp to be ordinarily used in rinsing the mouth, especially the latter solution; they may, however, be applied on the brush. The same is true of listerine.

Since, in rinsing the mouth, one does not keep the liquid in contact with the teeth longer than a minute, we need as an antiseptic mouthwash a substance which it capable of devitalizing the micro-organisms of the oral cavity in one minute or less. In my experiments for determining such substances, I have used the following method. I first prepared a tube containing a pure culture of one of the more important fungi of the human mouth, we may call this the *infecting tube;* second, a tube containing 0·5 c.c. of the antiseptic to be tested, we will call this the *antiseptic tube;* and third a number of tubes containing 5·0 c.c. of culture liquid or culture gelatine, these we will call the *culture tubes.*

A small drop or bead is conveyed from the infecting tube to the antiseptic tube on a loop of fine platinum wire, and then at intervals, varying from ¼ min. to 15 min., beads are conveyed from the antiseptic tube to each of the culture tubes in succession, one bead to each tube. These tubes are then kept at the temperature of the oral cavity. If the fungi were *all* devitalized by their passage through the antiseptic, the culture tubes remain clear, otherwise they become cloudy in a period varying from 7 to 60 hours.

These experiments should be doubly controlled: 1st, two culture tubes should be infected, using only sterilised water in the antiseptic tube; these two tubes should become cloudy in seven to ten hours, and if either of the other tubes become cloudy in the same time, it indicates that the antiseptic through which the fungi were passed had no effect whatever upon them.

The retardation in the appearance of the cloudiness will give a measure of the action of the antiseptic in those cases where a complete sterilization was effected. Second, the experiment should be repeated, using only sterilized water in both the infecting and antiseptic tubes. In this case the culture tubes should remain indefinitely clear. Should they become cloudy, it would indicate clumsy work on the part of the experimenter, and his experiments would be worthless. It is advisable to sterilise the air of the room before performing these experiments.

The results will be seen in the adjoined table:—

| Antiseptic. | Time of exposure necessary for Sterilization. |
|---|---|
| Salicylic Acid, 1—100 . . . | ¼ min. |
| Benzoic Acid, 1—100 . . . | ¼ " |
| Listerine . . . . . | ¼—½ min. |
| Salicylic Acid, 1—200 . . . | ½ " |
| Bichloride of Mercury, 1—2500 . | ½—¾ " |
| Benzoic Acid, 1—200 . . . | 1—2 " |
| Bichloride of Mercury, 1—5000 . | 2—5 " |
| Peroxide of Hydrogen, 10 per cent, . | 10—15 " |
| Carbolic Acid, 1—100 . . . | 10—15 " |
| Oil of Peppermint . . . (In agreeable strength for Wash.) | 10—15 " |
| Permanganate of Potash, 1—4000 | over 15 " |
| Boracic Acid, 1—50 . . . | " 15 " |
| Oil of Wintergreen (in agreeable strength" | 15 " |

It will be seen at a glance that of the substances tested in this series, only four are available for the prophylactic treatment of the human mouth. These are bichloride of mercury, salicylic acid, benzoic acid and listerine. Of these four I think the bichloride is without doubt the most effective, because its action continues longer.

In the attempt to sterilise the oral cavity a very serious difficulty is encountered in the fact that the antiseptic cannot in many cases be brought into contact with all portions of it. The free surfaces may become sterilised, but portions less exposed, especially the approximal surfaces of the teeth, fissures, cavities of decay, &c., escape the action of the antiseptic unless it may be kept for some length of time in the mouth. It is here that the bichloride of mercury (1 to 2500) possesses a decided advantage; after the body of the liquid has been put out of the mouth, the traces remaining continue their action until they have become diluted 120 times, this substance, in the proportion of 1 to 300,000, still having a retarding action upon the development of fungi. It appears also to be more penetrating than either of the other three available antiseptics. Unfortunately, however, it possesses one great disadvantage—its highly poisonous character. It seems, however, scarcely possible that any harm can result from its use in so dilute a form. The maximal dose of corrosive sublimate *pro die* is 0·1, and if we suppose that each time the mouth is washed with the 1 to 2500 solution (once daily), 0·5 c.c. of the solution gets into the system (a very high estimate), then it would be 500 days before the amount which may be administered in one day would be reached.

As just remarked, the possibility of any bad result seems almost excluded, and yet the element of certainty is lacking, and though I use the solution myself continually, often as strong as 1 to 1000, I hesitate to recommend it to my patients.

Considering the great interest which this question must possess for every dentist at least, I would recommend members of the profession to test the matter by experiments upon themselves and report the result. In no other way can a satisfactory solution of the question be arrived at. For self-experimentation I would advise the use of a solution of strength 1 to 1000, with sufficient oil of wintergreen to disguise the taste. It has, furthermore, been claimed that a solution of bichloride of mercury corrodes the teeth; this, however, needs confirmation.

As to salicylic acid, some say that it decalcifies the teeth, others deny that it has any effect of that kind. Buch (*Journal of Mat. Med.*, 1880), after having used a solution of salicylic acid, in strength 3 to 1000, for some weeks was obliged to discontinue its use, because he noticed "a curious feeling in his mouth, the teeth became softer, and their surfaces rough, through the formation of salicylate of lime." On the other hand, a chemist in Berlin has used a much stronger solution for over 10 years. His teeth when he began were in a deplorable state, but in a short time he succeeded in permanently arresting the progress of caries, of which there is now not a trace in his mouth, while the old cavities are brown and hard. No evidence whatever of any deleterious action up the teeth can be seen. It seems, however, fully well established that salicylic acid is a substance which must be used in the mouth with great caution, to say the least.

The somewhat weaker benzoic acid might be substituted for salicylic, provided it also should not prove to have an injurious effect upon the teeth.

Particularly noteworthy is the rapidity with which listerine acts. It appears to be one of the strongest and safest of the available antiseptic solutions. It owes its efficiency in all probability more to the boro benzoic acid which it contains than to the eucalyptus.

In comparing the two tables given above we notice what appears to be very great contradictions. Why does the peroxide of hydrogen, which in one series stands very high, fall so low in the other? and why should listerine, which is 40 times weaker than a 10 per cent. solution of peroxide of hydrogen, effect a sterilisation 30 times quicker? I can account for this striking difference only on the supposition that the rapidity with which a given antiseptic acts is by no means necessarily proportional to its ultimate strength. It is necessary first to all that the antiseptic pass through the cell membrane before it can act upon the protoplasm of the organism. This passage seems to take place very quickly in case of some substances, and comparatively slowly in case of others, although the latter, once through, may act more powerfully than the former. We must also bear in mind that the materials in the proportions given in the first table only prevent the development of the fungi ,while by the proportions in the second the complete devitalization is accomplished. There is a very great difference between the two. Many substances and conditions prevent by their mere presence the development of fungi without in the least rendering them incapable of development when they are brought into the proper medium. We need, of course, for most dental uses substances which produce complete sterilisation in a short time of contact.

It is furthermore an important fact that the substance which it is wished to sterilize may so act upon the antiseptic as to materially lessen or destroy its power. In this respect the permanganate of potash is very sensitive. Some antiseptics are liable to lose in strength with time. A preparation of peroxide of hydrogen from the best source in Berlin lost one-third in two months. I was particularly surprised and disappointed in the results obtained with this substance. I hoped to find in it a more effective and available antiseptic than any which we now possess, but in this I was completely deceived, its slowness of action unfitting it almost altogether for dental uses. It may act more quickly upon putre-factive organisms. This question I will determine by further experiments.—*Dental Record.*

